# Molecular targets and mechanisms of Sijunzi decoction in the treatment of Parkinson’s disease: evidence from network pharmacology, molecular docking, molecular dynamics simulation, and experimental validation

**DOI:** 10.3389/fphar.2024.1487474

**Published:** 2024-11-26

**Authors:** Yang Jiang, Wanfeng Wu, Le Xie, Yue Zhou, Kailin Yang, Dahua Wu, Wenfeng Xu, Rui Fang, Jinwen Ge

**Affiliations:** ^1^ Hunan Academy of Chinese Medicine, Changsha, Hunan, China; ^2^ Department of Gastroenterology, Hunan Provincial Hospital of Integrated Traditional Chinese and Western Medicine, Changsha, Hunan, China; ^3^ Department of Neurology, Hunan Provincial Hospital of Integrated Traditional Chinese and Western Medicine, Changsha, Hunan, China; ^4^ Department of Scientific Research, Hunan Academy of Chinese Medicine, Changsha, Hunan, China; ^5^ School of Integrated Chinese and Western Medicine, Hunan University of Chinese Medicine, Changsha, Hunan, China; ^6^ Hunan Provincial Hospital of Integrated Traditional Chinese and Western Medicine, Changsha, Hunan, China; ^7^ Department of Nephrology, The First Hospital of Hunan University of Chinese Medicine, Changsha, Hunan, China; ^8^ Institute of Clinical Pharmacology of Chinese Medicine, Hunan Academy of Chinese Medicine, Changsha, Hunan, China

**Keywords:** Sijunzi decoction, Parkinson’s disease, molecular docking, network pharmacology, molecular dynamics simulation

## Abstract

**Aim:**

To explore the molecular mechanism of Sijunzi Decoction (SJZD) in the treatment of Parkinson’s disease (PD) through the application of network pharmacology, molecular docking, and molecular dynamics simulations, complemented by experimental verification.

**Methods:**

The BATMAN-TCM, GeneCards, and DisGeNet databases were searched to screen the active components and therapeutic targets of SJZD. Cytoscape (3.7.1) was used to create a network diagram of the components and targets. The STRING platform was used to construct a protein-protein interaction (PPI) network. The Bioconductor database and RX64 (4.0.0) software were used to conduct Gene Ontology (GO) and Kyoto Encyclopedia of Genes and Genomes (KEGG) enrichment analysis on the core target genes. The binding sites and binding energies between SJZD active components and the target were analyzed by molecular docking and dynamic simulation. Finally, the therapeutic effect and mechanism of SJZD were verified by Cell Counting Kit-8 (CCK-8) and Western blotting (WB).

**Results:**

This research identified 188 active compounds in SJZD, 1568 drug targets, 2069 PD targets, and 451 intersection targets related to PD. According to network analysis, Adenosine Triphosphate, Tridecanoic Acid, Hexadecanoic Acid, Pentadecanoic Acid, and Adenosine were identified as the core components of SJZD in the treatment of PD. The five targets with the highest Degree values in the PPI network were AKT1, INS, TNF, IL-6, and TP53. The GO and KEGG enrichment analyses, in turn, determined that the administration of SJZD for the treatment of PD may engage processes such as xenobiotic stimulation and biological stimulus response. Furthermore, AGE-RAGE and cAMP signaling pathways related to diabetic complications may be involved. Molecular docking and kinetic simulations showed that IL-6 and AKT1 bind best to Adenosine. Experimental results showed that SJZD significantly reduced 6-OHDA-induced apoptosis of SH⁃SY5Y cells by activating the PI3K/AKT signaling pathway and regulating the expression of apoptosis factors such as Bcl⁃2 and Bax.

**Conclusion:**

SJZD is essential in the processes of apoptosis and neuronal protection, acting through various components that target multiple pathways. Notably, the PI3K/AKT pathway is a verified SJZD-PD target, providing a reference for clinical precision drug use for PD.

## 1 Introduction

Parkinson’s disease (PD) is a common neurodegenerative disease affecting middle-aged and older adults, with the majority of diagnoses occurring in people over 60 years old (Arena et al., 2021). Its clinical symptoms include motor and non-motor symptoms. The typical motor symptoms include static tremor, muscle rigidity, motor delay, and postural instability, while the non-motor symptoms typically comprise mood disorders, cognitive difficulties, and sleep disturbance (Ip et al., 2014). Epidemiological studies show that, in Europe and the United States, the prevalence of PD among people aged over 60 is 1%, while the rate among those aged over 80 is higher than 4% (Parkinson’s Disease and movement Disorders Group of Neurology Branch of Chinese Medical Association and Parkinson’s Disease and Movement Disorders Group of Neurology Branch of Chinese Medical Doctor Association, 2020). The incidence of PD among people aged 65 years and older in China is 1.7%, and continues to rise. Previous research estimates that by the year 2030, the number of individuals with PD in China could approach 5 million, representing over fifty percent of the worldwide PD population (Parkinson’s Disease and movement Disorders Group of Neurology Branch of Chinese Medical Association and Parkinson’s Disease and Movement Disorders Group of Neurology Branch of Chinese Medical Doctor Association, 2020). Its onset is believed to be caused by mitochondrial dysfunction and oxidative stress within dopamine neurons in the substantia nigra, resulting in their death and leading to a dopamine deficiency in the striatum (Venkateshappa et al., 2012). This disease is generally diagnosed at a relatively advanced stage when a considerable number of dopamine-producing neurons in the brain have already degenerated, creating a need for efficacious modes of treatment. Levodopa remains the most widely utilized pharmacological therapy for PD symptomatic treatment (Neo et al., 2020). Nevertheless, prolonged administration of levodopa is associated negative side effects, including motor fluctuations and dyskinesias (Bogetofte et al., 2020).

Traditional Chinese medicine (TCM) is a viable option for the treatment of PD due to its recognized low incidence of side effects and its comprehensive treatment strategy that targets multiple pathways and mechanisms. TCM has demonstrated efficacy in enhancing mitochondrial function, regulating autophagy and neurotransmitter levels, and addressing oxidative stress, inflammation, and apoptosis (Chen et al., 2022). Sijunzi Decoction (SJZD) is as a classic prescription for the treatment of spleen deficiency syndrome. It has long been used to treat various gastrointestinal disorders, including chronic gastritis and ulcerative colitis without side effects. Notably, recent research has found that four herbs [*Panax ginseng C. A. Mey.* (RenShen), *Atractylodes macrocephala Koidz* (BaiZhu), *Poria cocos (Schw.) Wolf* (FuLing), and *Glycyrrhizae Radix Et Rhizoma Praeparata Cum Melle* (GanCao)] found in SJZD have been clinically used to treat neurodegenerative diseases and have shown both neuroprotective and antioxidant properties (Chen et al., 2022). Moreover, components of SJZD have been identified to reverse inflammation in neurodegenerative diseases such as PD (Ye et al., 2014). However, little research has gone into establishing these characteristics and their underlying mechanisms. The involvement of the PI3K/AKT pathway in the formation of two special pathological structures in PD has been established (Yang et al., 2020). It regulates neurotoxicity and facilitates neuronal survival through various substrates such as Bcl⁃2 and Bax (Kumari et al., 2023). Therefore, using drugs that influence the activity of this pathway and its substrates is a viable option for PD treatment. Though SJZD has been used in the treatment of PD (Chen et al., 2019; Liu et al., 2021), its pharmacotherapeutic mechanism and active compounds have not been well studied and established.

**FIGURE 1:**
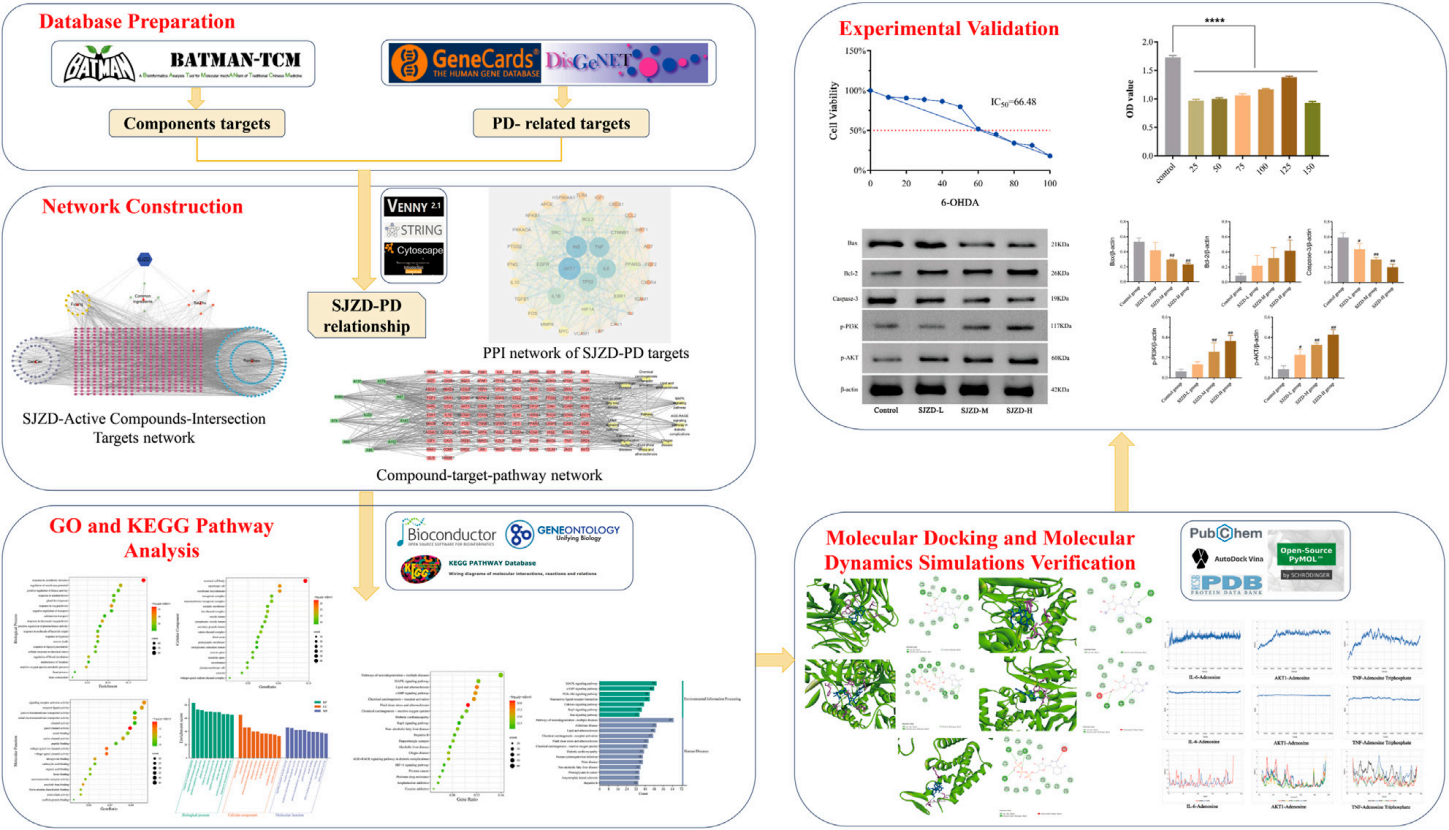
Flowchart of the network pharmacological investigation strategy of SJZD in the treatment of PD. Five parts include database preparation, network construction, GO and KEGG pathway analysis, molecular docking and dynamics simulations verification, and experimental verification.

Network pharmacology is an interdisciplinary approach that integrates systems biology, computer biology, and various other technologies to explore the relationship between TCM therapies and diseases (Zhang et al., 2019). Molecular docking is a computational technique of bioinformatics that utilizes the characteristics of receptors and the interaction between receptors and drug molecules to design drugs. It primarily analyzes the binding patterns and affinity between ligands and receptors. Diversely, molecular dynamics simulation is used to investigate the dynamic behavior of biological macromolecules such as proteins and DNA. This technique can simulate the motion state of biological macromolecules in three-dimensional space, uncover the mechanism of biological function, and identify small molecules and potential targets (Kumar et al., 2022; Bhardwaj and Purohit, 2022; Singh et al., 2022). This research utilized network pharmacology, molecular docking, molecular dynamics simulation, and experimental verification to explore the potential targets, pathways, and mechanisms of action of SJZD in treating PD, thus providing a foundation for clinical rational drug use. A flowchart of this study is shown in Figure 1.

## 2 Materials and methods

### 2.1 Screening effective components and targets of SJZD

The BATMAN-TCM database (Liu et al., 2016) was used to identify the effective components and targets of SJZD through screening using BaiZhu, FuLing, GanCao, and RenShen as the search keywords. The criteria for screening had a correlation score of more than 20 and a *p*-value of less than 0.05, and the target names were standardized via the Uniprot database. Any duplicate targets were removed and merged into the target database.

### 2.2 Prediction of targets associated with PD

After searching for “Parkinson’s Disease” on both the DisGeNet database (https://www.disgenet.org/) (Zhang et al., 2018) and GeneCards database (http://www.genecards.org/) (Guo et al., 2020), we created a database of disease targets related to PD by eliminating any duplicates and compiling the relevant genes.

### 2.3 Establishing “SJZD-active compounds-intersection targets” network

By intersecting the SJZD and PD target databases, we obtained the intersection target of “SJZD-PD” which was then visualized using the VENNY diagram (https://bioinfogp.cnb.csic.es/tools/venny). Subsequently, the intersection targets, active components, and drug names were imported into Cytoscape 3.7.1 software to construct and visualize the “SJZD-Active Compounds-Intersection Targets” network. Finally, the core drug targets were identified based on the Degree value.

### 2.4 Establishing “protein-protein interaction (PPI)” network

The target obtained from “SJZD-PD” was imported into the String database (https://www.string-db.org/) (Szklarczyk et al., 2015). A PPI network was obtained by limiting the study species to “*Homo sapiens*” and setting the minimum threshold to “medium confidence (0.400)” (Wang et al., 2017), and core targets were screened by calculating the Degree value. The results were then visually optimized using Cytoscape 3.7.1.

### 2.5 GO and KEGG enrichment analyses

Utilizing the Bioconductor database and the RX64 4.0.0 software, Gene Ontology (GO) and Kyoto Encyclopedia of Genes and Genomes (KEGG) enrichment analyses were conducted on core target genes. This analysis focused on three categories: biological process (BP), cell composition (CC), and molecular function (MF) (Ye et al., 2021). We used a free online platform (http://www.bioinformatics.com.cn) to visualize the results.

### 2.6 Molecular docking

Using the PubChem database (https://pubchem.ncbi.nlm.nih.gov/), the core compounds in the network diagram of “SJZD-Active Compounds-Intersection Targets” were obtained in SDF file format. OpenBabel software was used to convert the SDF file into mol2 format, and AutoDock Tool software (Trott and Olson, 2010) was used to perform hydrogenation, ligand detection, and other operations before saving it in pdbqt format. The core protein structure was obtained from the PDB database (http://www.rcsb.org/), and Pymol software (Zhu and Hou, 2020) was used to remove its ligands and water molecules. AutoDock Vina software was then used to link the protein structure and compound molecules for molecular docking, and the results were visualized and analyzed using Discover Studio software 4.5.

### 2.7 Molecular dynamics simulation

Molecular dynamics simulations were conducted on the top three complexes with the highest molecular docking scores using the Amber18 software package. The protein was assigned the ff14SB force field parameter, while the small molecule ligand was assigned the gaff universal force field parameter and had its AM1-BCC atomic charge calculated using the ANTECHAMBER module. The complexes were loaded into the TLAP module, with hydrogen atoms and antagonistic ions added to neutralize charges. The TIP3P explicit water model was selected, and periodic boundary conditions were set. The simulation workflow included four steps: energy minimization, heating, equilibration, and production kinetics simulation. After energy minimization of the heavy atoms of the proteins (and small molecules) in 10,000 steps, constraints were relaxed, and an additional 10,000 steps of energy optimization were conducted on the entire system. The system was then slowly heated to 300 K throughout 50 ps, followed by equilibration at 50 ps under the NPT ensemble. Finally, a molecular dynamics simulation of 200 ns was conducted under the NPT ensemble, with a time step of 2 fs. Trajectory data was saved every 40 ps, and correlation analysis was performed using the CPPTRAJ module. The binding free energy of the ligands and proteins was calculated using the MMPBSA.py module.

### 2.8 Cell culture

Human SH⁃SY5Y neuroblastoma cells were purchased from Wuhan Punosai Life Technology Co., LTD, and cultured in DMEM/F12 medium (AW-M006, Abiowell, Hunan, China) containing 15% fetal bovine serum (FBS, AWC0227a, Abiowell, Hunan, China) and 1% penicillin-streptomycin (AWH0529a, Abiowell, Hunan, China). The cells were maintained in a constant temperature incubator with 5% CO_2_ at 37°C. When the cells reached a confluence of 80%–90%, they were digested with trypsin. This digestion process was subsequently halted using a complete medium. Following centrifugation, the cells were suspended and transferred to a new culture bottle. Cellular experiments were conducted on an ultra-clean workbench (YT-CJ-2NB, YATAIKELONG, Beijing, China). During the cell culture process, all containers were sterilized in a high-temperature autoclave, and ultraviolet sterilization was used for the ultra-clean workbench.

### 2.9 Screening of the optimal 6-OHDA or SJZD concentrations and cell viability analysis

Logarithmic SH-SY5Y cells were inoculated on 96-well plates (703001, NEST, Jiangsu, China) with a density of 5 × 10^3^ cells/well. After the cells adhered to the walls of the plates, they were treated with different concentrations of 6-OHDA (H4381, Sigma-Aldrich, St. Louis, MO, United States) (0, 10, 20, 30, 40, 50, 60, 70, 80, 90, 100 µM) and incubated at 37°C in 5% CO_2_ for 24 h. Thereafter, 70 µM was determined as the optimal concentration for further analysis. Under the same incubation conditions, SH-SY5Y cells treated with 70 µM 6-OHDA were exposed to different concentrations of SJZD (25, 50, 75, 100, 125, and 150 μg/mL), which was purchased from Hunan Provincial Hospital of Integrated Traditional Chinese and Western Medicine. SH-SY5Y cells and 6-OHDA-treated SH-SY5Y cells were incubated with 10 μL Cell Counting Kit-8 (CCK-8) buffer (Dojindo, Japan) for 4 h to determine half maximal inhibitory concentration (IC_50_) (Supplementary Figure S1) and optimal SJZD concentration, respectively. The absorbance values for each hole were measured at a wavelength of 450 nm using a multifunctional enzyme label analyzer (Huisong, Shenzhen, China).

### 2.10 Western blot analysis

To study the effect of different doses of SJZD, the cells were divided into the SJZD-L group (75 μg/mL), the SJZD-M group (100 μg/mL), the SJZD-H group (125 μg/mL), and the control group. RIPA lysate was added into the cells for protein extraction and centrifuged at 12000 rpm for 15 min at 4°C. The protein concentration was determined using the BCA method, with absorbance measured at a wavelength of 562 nm using an enzymoscope, followed by constructing a standard curve. The protein was transferred onto the nitrate cellulose membrane and blocked with 5% skim milk powder at room temperature for 1 h. The membranes were incubated with Bax (1:2000, 50599-2-Ig, Proteintech, United States), Bcl-2 (1:500, 26593-1-AP, Proteintech, United States), Caspase-3 (1:500, 19677-1-AP, Proteintech, United States), p-PI3K (1:500, Bs-5570R, Proteintech, United States), and p-AKT (1:500, 66444-1-Ig, Proteintech, United States) at 4°C overnight. The film was then washed three times with TBST (Biosharp, Anhui, China), and incubated with a secondary antibody (1:5000, AWH0529a, Abiowell, Hunan, China) at room temperature for 1 h. Finally, the washed film was developed with ECL luminescent reagent (ThermoFisher Scientific, Shanghai, China) away from light. ImageJ software (NIH, Bethesda, MD) was used to analyze the gray values of the target protein bands. β-actin (1:500, Proteintech, United States) was used as the loading control.

### 2.11 Statistical analysis

SPSS 26.0 software was used for statistical analysis, and the data were expressed as mean ± standard deviation $\left(\bar{x}\pm s\right)$ . For comparison of data among multiple groups, One-way ANOVA was used when the variance was homogeneous, and LSD method was used for multiple comparisons after the event. Dunnett’s T3 method is used when variance is uneven. The statistical significance level was set as *p* < 0.05.

## 3 Results

### 3.1 Effective compounds and targets of SJZD

We obtained 5 active compounds from BaiZhu, 18 from FuLing, 57 from GanCao, and 112 from RenShen. After eliminating duplicates, a total of 188 active compounds were identified. Table 1 shows the effective components of each TCM. We found 82 targets for BaiZhu, 654 for FuLing, 1004 for GanCao, and 4813 for RenShen. After eliminating duplicates, the total number of drug targets was 1568.

**TABLE 1:** Effective compounds of SJZD.

ID	TCM	Effective compounds	ID	TCM	Effective compounds
A1	BaiZhu	4-Ethoxycarbonyl-2-Quinolone	A95	RenShen	Beta-Bisabolene
A2	BaiZhu	Atractylenolide Iii	A96	RenShen	Beta-Elemene
A3	BaiZhu	Beta-Eudesmol	A97	RenShen	Beta-Humulene
A4	BaiZhu	Hinesol	A98	RenShen	Beta-Santalol
A5	BaiZhu	Jurubine	A99	RenShen	Beta-Selinene
A6	FuLing	10-Hydroxyacetylbaccatin Vi	A100	RenShen	Bicyclogermacrene
A7	FuLing	Beta-Amyrin Acetate	A101	RenShen	Biotin
A8	FuLing	Dehydroeburicoicacid	A102	RenShen	Calarene
A9	FuLing	Eburicol	A103	RenShen	Campesterol
A10	FuLing	Ergosterol	A104	RenShen	Chikusetsusaponin Iii
A11	FuLing	Ergotamine	A105	RenShen	Chikusetsusaponin Iv
A12	FuLing	Hydrangeic Acid	A106	RenShen	Citronellal
A13	FuLing	Lauric Aldehyde	A107	RenShen	Dauricine
A14	FuLing	O-Acetylpachymic Acid-25-Ol	A108	RenShen	Delta-Elemene
A15	FuLing	Pachymic Acid	A109	RenShen	Delta-Guaiene
A16	FuLing	P-Hydroxybenzyl Alcohol	A110	RenShen	Deoxyharringtonine
A17	FuLing	Poricoic Acid B	A111	RenShen	Dianthramine
A18	FuLing	Turanose	A112	RenShen	Dibutyl Oxalate
A19	FuLing	Undecan-2-Ol	A113	RenShen	Dibutyl Phthalate
A20	GanCao	18alpha-Glycyrrhetinic Acid	A114	RenShen	Elemicin
A21	GanCao	18beta-Glycyrrhetinic Acid	A115	RenShen	Epsilon-Cadinene
A22	GanCao	2,4,4′-Trihydroxychalcone	A116	RenShen	Gamma-Selinene
A23	GanCao	2,5-Dihydroxymethyl-3,4-Dihydroxypyrrolidine	A117	RenShen	Gamma-Sitosterol
A24	GanCao	2-Methyl-1,3,6-Trihydroxyanthraquinone	A118	RenShen	Ginsenol
A25	GanCao	3-Hydroxyglabrol	A119	RenShen	Ginsenoside F1
A26	GanCao	3′-Methoxyglabridin	A120	RenShen	Ginsenoside La
A27	GanCao	4′-O-Methylglabridin	A121	RenShen	Ginsenoside Rb1
A28	GanCao	5,6,7,8-Tetrahydro-4-Methylquinoline	A122	RenShen	Ginsenoside Rb2
A29	GanCao	8-Methyl-10-Hydroxylycoctonine	A123	RenShen	Ginsenoside Rc
A30	GanCao	Alpha-Trihydroxy Coprostanic Acid	A124	RenShen	Ginsenoside Rd
A31	GanCao	Corylifolinin	A125	RenShen	Ginsenoside Re
A32	GanCao	Dimethyl Sebacate	A126	RenShen	Ginsenoside Rg1
A33	GanCao	Ethyl-N-Buthy-Uralsaponin A Esters	A127	RenShen	Ginsenoside Rg3
A34	GanCao	Gancaonin E	A128	RenShen	Ginsenoside Rh2
A35	GanCao	Gancaonin F	A129	RenShen	Ginsenoside-La
A36	GanCao	Gancaonin I	A130	RenShen	Ginsenoside-Rb1
A37	GanCao	Glisoflavanone	A131	RenShen	Ginsenoside-Rb2
A38	GanCao	Gloeosteretriol	A132	RenShen	Ginsenoside-Rc
A39	GanCao	Glycyrin	A133	RenShen	Ginsenoside-Rd
A40	GanCao	Glycyrol	A134	RenShen	Ginsenoside-Re
A41	GanCao	Glycyrrhetinic Acid	A135	RenShen	Ginsenoside-Rh1
A42	GanCao	Glycyrrhetol	A136	RenShen	Guanosine
A43	GanCao	Glycyrrhizic Acid	A137	RenShen	Hexadecanoic Acid
A44	GanCao	Glycyrrhizin	A138	RenShen	Humulene
A45	GanCao	Glyuranolide	A139	RenShen	Kaempferol
A46	GanCao	Glyyunnanprosapogenin D	A140	RenShen	Malonylginsenoside Rc
A47	GanCao	Hispaglabridin A	A141	RenShen	Malonylginsenoside Rd
A48	GanCao	Hispaglabridin B	A142	RenShen	Maltose
A49	GanCao	Isoglycyrol	A143	RenShen	Malvic Acid
A50	GanCao	Isoliensinine	A144	RenShen	Mannose
A51	GanCao	Isoliquiritigenin	A145	RenShen	Menthyl Acetate
A52	GanCao	Isoramanone	A146	RenShen	Methyl Palmitate
A53	GanCao	Isotrilobine	A147	RenShen	N-Octane
A54	GanCao	Licobenzofuran	A148	RenShen	Notoginsenoside R1
A55	GanCao	Licocoumarone	A149	RenShen	Notoginsenoside R2
A56	GanCao	Licoricesaponin C2	A150	RenShen	Notoginsenoside R4
A57	GanCao	Licoricesaponine A3	A151	RenShen	Octanal
A58	GanCao	Licoricesaponine C2	A152	RenShen	Palmitoleic Acid
A59	GanCao	Licoricesaponine D3	A153	RenShen	Panacon
A60	GanCao	Licoricesaponine F3	A154	RenShen	Panasinsanol A
A61	GanCao	Licoricesaponine G2	A155	RenShen	Panasinsanol B
A62	GanCao	Licoricesaponine J2	A156	RenShen	Panaxadiol
A63	GanCao	Licoricidin	A157	RenShen	Panaxatriol
A64	GanCao	Licorisoflavan A	A158	RenShen	Pandamine
A65	GanCao	Liquiritigenin-7,4′-Diglucoside	A159	RenShen	Patchouli Alcohol
A66	GanCao	Methyl Linoleate	A160	RenShen	Pentadecanoic Acid
A67	GanCao	Methyl-24-Hydroxyglycyrrhetate	A161	RenShen	Protopanaxadiol
A68	GanCao	Methylglyoxal	A162	RenShen	Protopanaxatriol
A69	GanCao	Monoammonium Glycyrrhizinate	A163	RenShen	Protopine
A70	GanCao	Narwedine	A164	RenShen	Pseudoginsenoside F11
A71	GanCao	Neohancoside A	A165	RenShen	Pseudohypericin
A72	GanCao	Neoliquiritin	A166	RenShen	Putrescine
A73	GanCao	Phaseollinisoflavan	A167	RenShen	Pyrrole-2-Aldehyde
A74	GanCao	Ruvoside	A168	RenShen	Raffinose
A75	GanCao	Tetrahydroharmine	A169	RenShen	Ramalic Acid
A76	GanCao	Urea	A170	RenShen	Rhamnose
A77	RenShen	12-O-Nicotinoylisolineolone	A171	RenShen	Riboflavine
A78	RenShen	16-Oxoseratenediol	A172	RenShen	Spermidine
A79	RenShen	1-Heptadecanol	A173	RenShen	Spermine
A80	RenShen	1-Tetradecanol	A174	RenShen	Stigmasterol
A81	RenShen	2,5-Dimethyl-7-Hydroxy Chromone	A175	RenShen	Sucrose
A82	RenShen	20(R)-Ginsenoside Rg3	A176	RenShen	Tauremisin
A83	RenShen	20(S)-Protopanaxadiol	A177	RenShen	Tetradecane
A84	RenShen	2-Heptadecanone	A178	RenShen	Trans-Caryophyllene
A85	RenShen	3,5-Dimethyl-4-Methoxybenzoic Acid	A179	RenShen	Tridecanoic Acid
A86	RenShen	Adenosine	A180	RenShen	Uridine
A87	RenShen	Adenosine Triphosphate	A181	RenShen	Vitamin B1
A88	RenShen	Alpha-Cadinol	A182	RenShen	Vitamin B12
A89	RenShen	Alpha-Farnesene	A183	RenShen	Widdrol
A90	RenShen	Alpha-Guriunene	A184	RenShen	Xylose
A91	RenShen	Aposcopolamine	T1	Common components	20-Hexadecanoylingenol
A92	RenShen	Aposiopolamine	T2	Common components	Adenine
A93	RenShen	Araloside A	T3	Common components	Choline
A94	RenShen	Argininyl-Fructosyl-Glucose	T4	Common components	Chrysanthemaxanthin

### 3.2 PD-associated targets and intersection target of “SJZD-PD”

**FIGURE 2:**
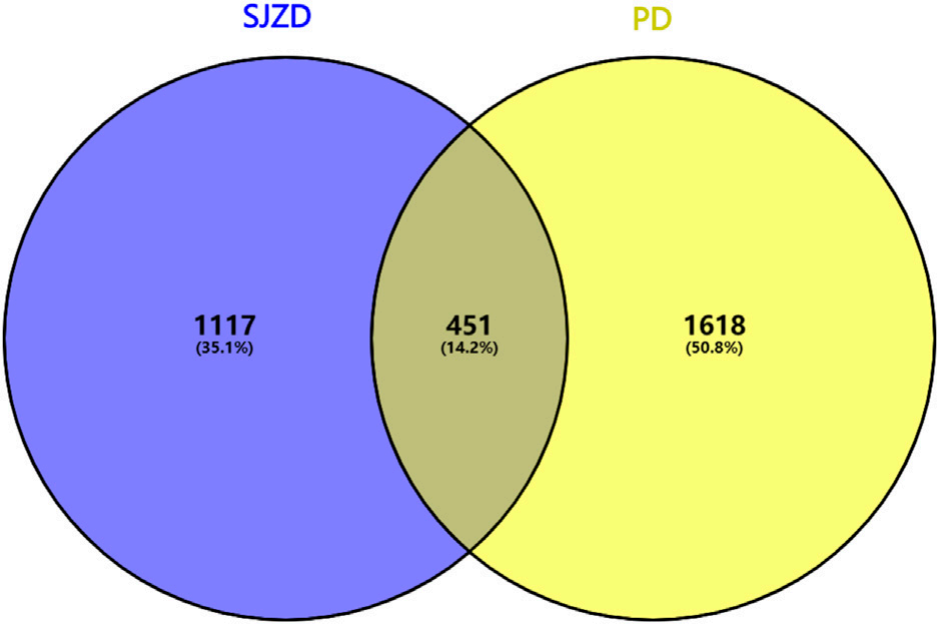
Venn diagram of potential SJZD target for PD. The blue on the left signifies the ambition of SJZD, the yellow on the right represents the objective of PD, and the middle part illustrates the shared targets of the two.

Through Genecard database (score ≥10) screening, 1853 targets were identified, with a further 497 DisGeNet (score ≥0.05) targets identified, resulting in a total of 2069 disease target genes after duplicates were removed. Utilizing PERL software, 451 intersection targets of “SJZD-PD” were identified and visualized through VENNY plots (Figure 2).

### 3.3 “SJZD-Active compounds-intersection targets” network and key effective compounds

**FIGURE 3:**
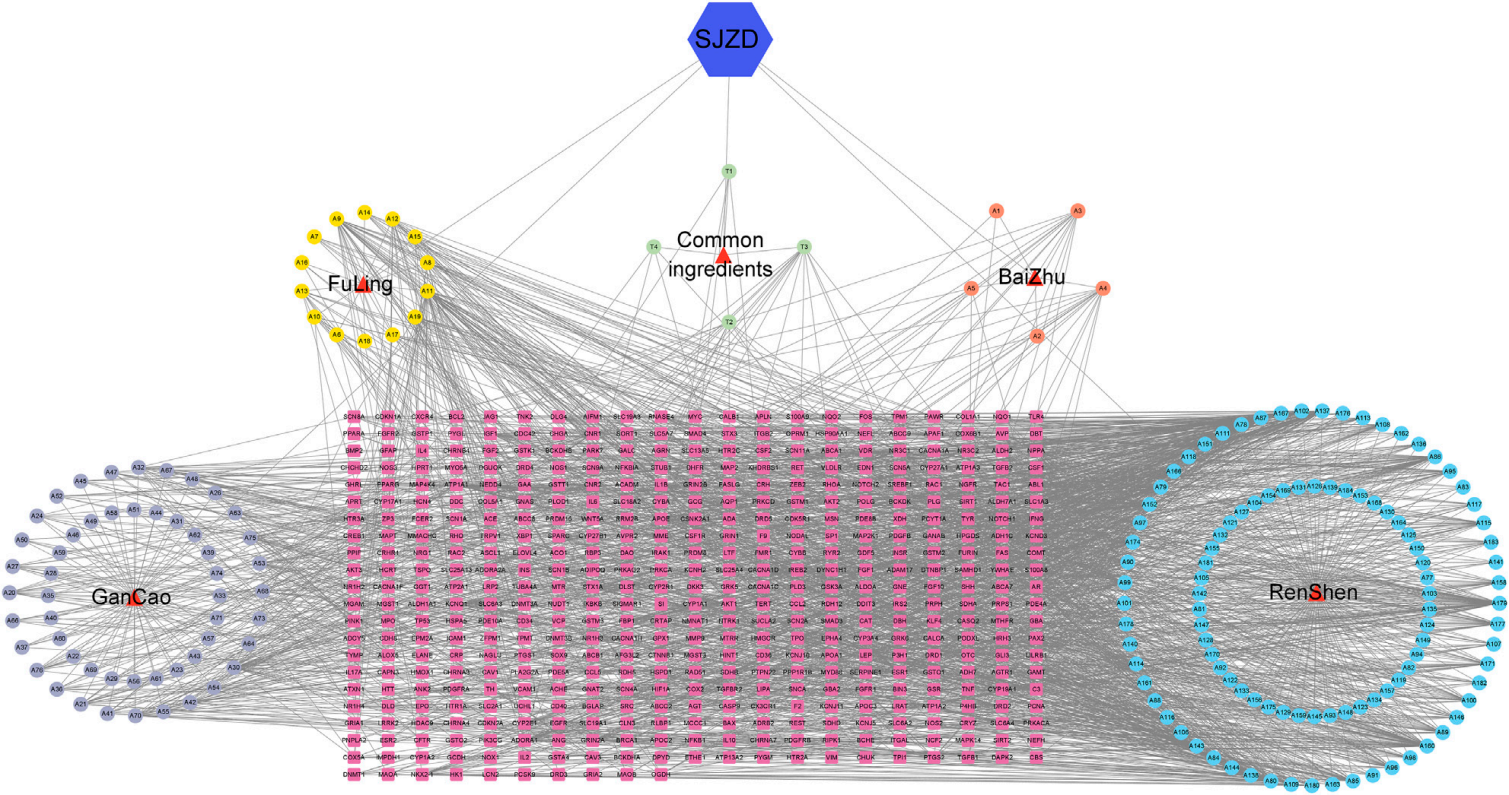
“SJZD-Active Compounds-Intersection Targets” network. The red triangles represent the 4 herbs in SJZD. The purple, yellow, orange, and blue circle nodes stand for GanCao, FuLing, BaiZhu, and RenShen compounds, respectively. The pink square nodes represent the predicted targets, and the edges represent the interactions between the compounds and the targets.

By uploading active compounds and common targets into Cytoscape 3.7.1, a “SJZD-Active Compounds-Intersection Targets” network comprising 635 nodes and 2341 edges (Figure 3) was created. The network provides insight into the complex effects of active compounds in SJZD on PD. Ranked from highest to lowest, Adenosine Triphosphate, Tridecanoic Acid, Hexadecanoic Acid, Pentadecanoic Acid, and Adenosine were the top five Degree values (Table 2). These are thought to be the primary active components of SJZD for treating PD.

**TABLE 2:** Basic information on the top five degrees of the effective compounds in SJZD.

TCM	ID	Mol name	Degree
RenShen	A87	Adenosine Triphosphate	110
RenShen	A179	Tridecanoic Acid	72
RenShen	A137	Hexadecanoic Acid	71
RenShen	A160	Pentadecanoic Acid	71
RenShen	A86	Adenosine	55

### 3.4 PPI network

**FIGURE 4:**
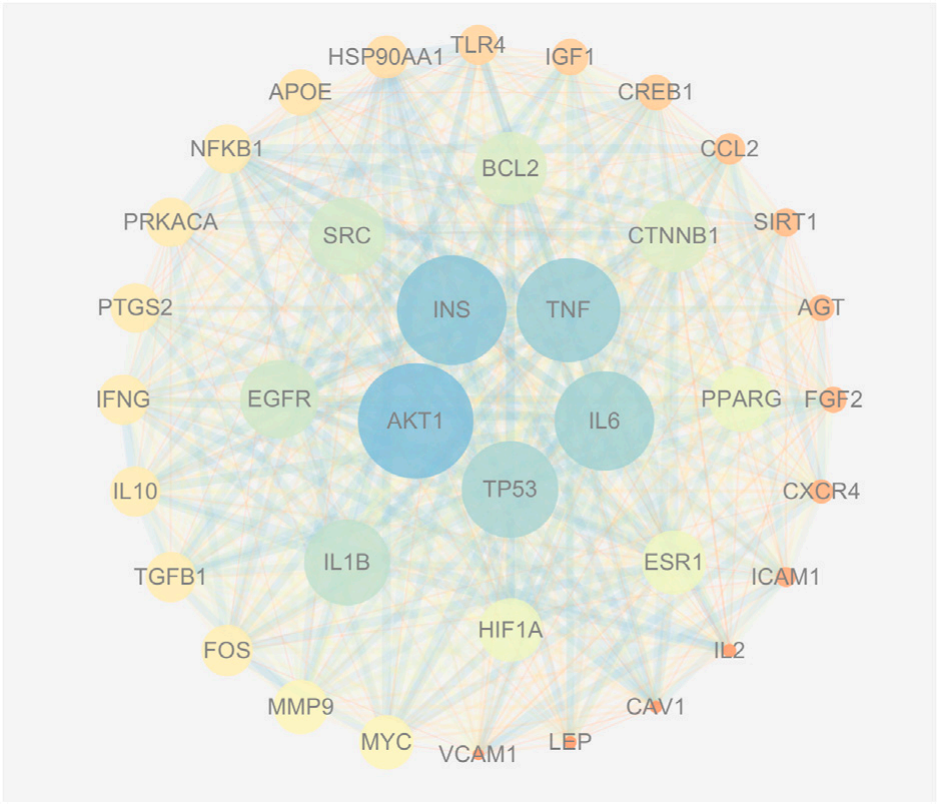
PPI network (Degree >60). As the Degree value increases, the circle’s color becomes bluer and its area larger; conversely, a decrease in Degree value results in a redder color and a smaller size. Similarly, a higher Combined score value produces a thicker connection between two points with a bluer hue, while a lower Combined score value produces a thinner connection with a redder shade.

**FIGURE 5:**
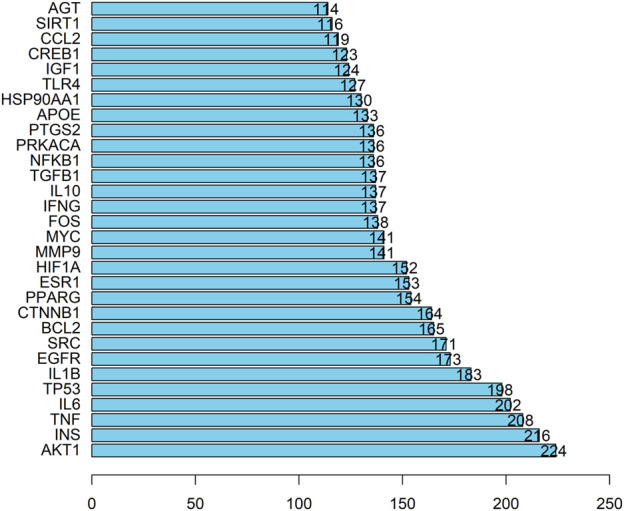
Top 30 targets. The Degree value is plotted on the horizontal axis, and the gene name is plotted on the vertical axis.

The PPI network consisting of 451 intersection targets included 448 nodes and 9753 edges, with an average node degree of 43.5 and an average local clustering coefficient of 0.483 (Figure 4). The target is represented by a circular node, and the larger the node, the higher the degree value, and the more important the target. Using R X64 4.0.0, a bar chart of the top 30 target genes was generated (Figure 5). In order of Degree, the top 5 were AKT1 (Degree = 224), INS (Degree = 216), TNF (Degree = 208), IL-6 (Degree = 202), and TP53 (Degree = 198). Therefore, these 5 targets can be considered the most important core targets of SJZD in the treatment of PD, warranting further study.

### 3.5 GO enrichment analysis

**FIGURE 6:**
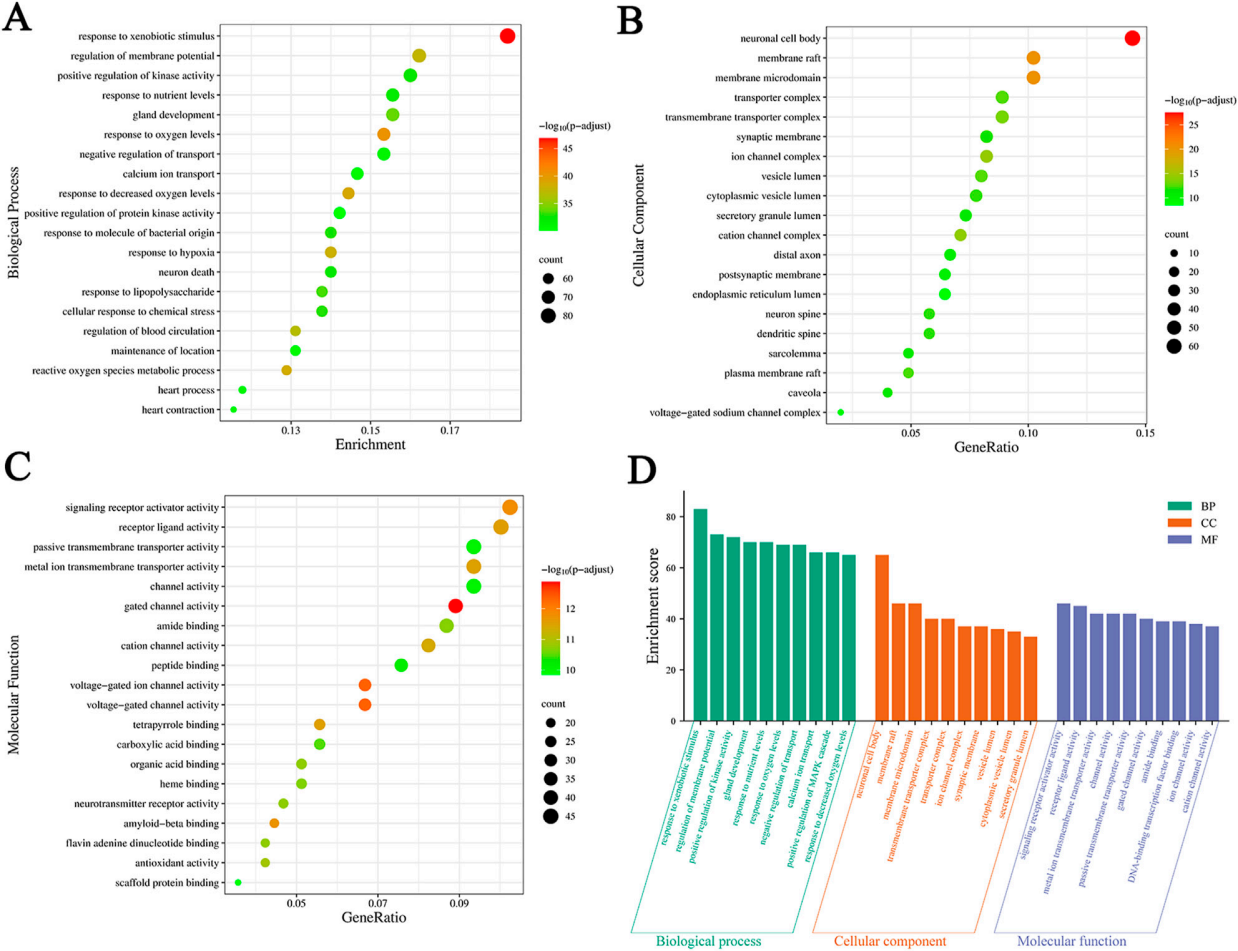
Results of GO enrichment analysis. **(A–C)** Bubble charts of the top 20 significant enrichment entries in BP, CC, and MF. The *X*-axis represents the proportion of genes of interest in the entries, and the *Y*-axis represents each entry. The color and size of each bubble represent the adjusted *p*-value and the gene counts, respectively. **(D)** The top 10 BP, CC, and MF entries of GO enrichment analysis are green, orange, and purple bars, respectively.

We conducted a GO enrichment analysis on 451 common targets to further investigate the mechanism of SJZD in treating PD. A total of 4211 statistically significant entries were identified in three categories, including 3726 entries for BP, 200 for CC, and 285 for MF. The analysis results are presented as bubble charts, with the redder colors indicating lower adjusted *p* values and higher enrichment of the GO entries (Figures 6A–C). Furthermore, the top 10 entries with the highest gene counts in each category are presented in Figure 6D and Table 3. These entries were mainly associated with various biological processes such as response to xenobiotic stimuli, response to oxygen levels, response to reduced oxygen content, reactive oxygen species metabolism, and response to hypoxia; cellular components such as neuronal cell bodies, membrane rafts, membrane microdomains, ion channel complexes, and cation channel complexes; and molecular functions such as gated ion channel activity, voltage-gated ion channel activity, voltage-gated channel activity, signal receptor activator activity, and amyloid protein-β binding.

**TABLE 3:** Top 10 enriched entries in GO enrichment analysis.

Classification	ID	GO	*p*-value	Number of genes
BP	GO:0009410	response to xenobiotic stimulus	2.23 × 10^−51^	83
BP	GO:0070482	response to oxygen levels	1.65 × 10^−44^	69
BP	GO:0036293	response to decreased oxygen levels	1.08 × 10^−42^	65
BP	GO:0072593	reactive oxygen species metabolic process	2.72 × 10^−42^	58
BP	GO:0001666	response to hypoxia	1.05 × 10^−41^	63
BP	GO:0042391	regulation of membrane potential	3.99 × 10^−41^	73
BP	GO:1903522	regulation of blood circulation	1.88 × 10^−40^	59
BP	GO:0048732	gland development	1.64 × 10^−37^	70
BP	GO:0032496	response to lipopolysaccharide	6.23 × 10^−37^	62
BP	GO:0062197	cellular response to chemical stress	1.82 × 10^−36^	62
CC	GO:0043025	neuronal cell body	6.23 × 10^−31^	65
CC	GO:0045121	membrane raft	1.89 × 10^−23^	46
CC	GO:0098857	membrane microdomain	2.15 × 10^−23^	46
CC	GO:0034702	ion channel complex	2.85 × 10^−17^	37
CC	GO:0034703	cation channel complex	6.56 × 10^−17^	32
CC	GO:1902495	transmembrane transporter complex	5.39 × 10^−16^	40
CC	GO:0031983	vesicle lumen	4.46 × 10^−15^	36
CC	GO:1990351	transporter complex	5.16 × 10^−15^	40
CC	GO:0044853	plasma membrane raft	8.55 × 10^−15^	22
CC	GO:0060205	cytoplasmic vesicle lumen	2.12 × 10^−14^	35
MF	GO:0022836	gated channel activity	1.54 × 10^−16^	40
MF	GO:0005244	voltage-gated ion channel activity	1.60 × 10^−15^	30
MF	GO:0022832	voltage-gated channel activity	1.60 × 10^−15^	30
MF	GO:0030546	signaling receptor activator activity	7.54 × 10^−15^	46
MF	GO:0001540	amyloid-beta binding	1.16 × 10^−14^	20
MF	GO:0048018	receptor ligand activity	2.01 × 10^−14^	45
MF	GO:0046906	tetrapyrrole binding	2.42 × 10^−14^	25
MF	GO:0046873	metal ion transmembrane transporter activity	2.86 × 10^−14^	42
MF	GO:0005261	cation channel activity	4.22 × 10^−14^	37
MF	GO:0016209	antioxidant activity	1.48 × 10^−13^	19

### 3.6 KEGG pathway enrichment analysis

**FIGURE 7:**
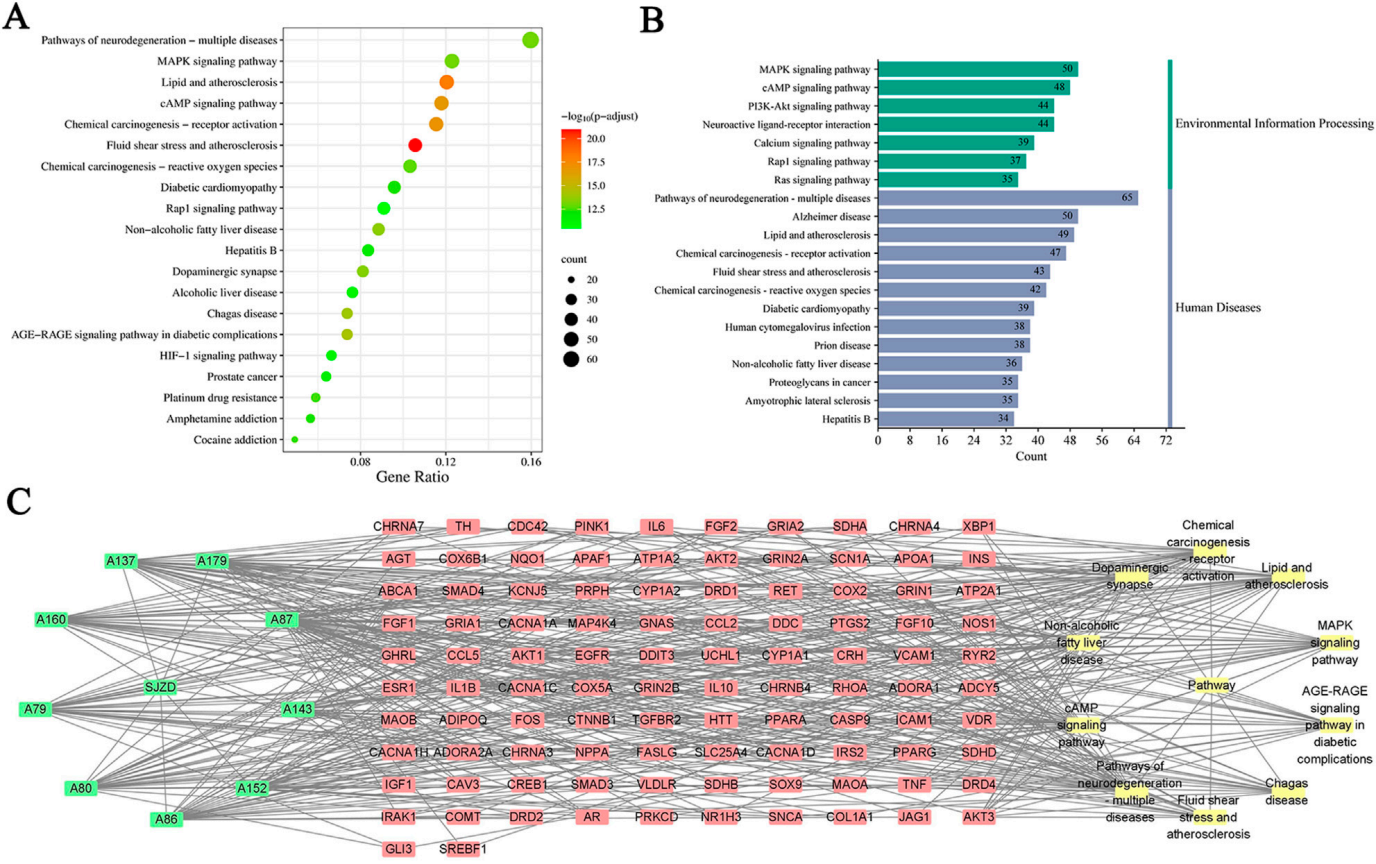
Results of KEGG enrichment analysis. **(A)** The bubble chart of the top 20 enrichment pathways based on KEGG enrichment analysis. **(B)** The KEGG type of the top 20 enrichment pathways obtained from KEGG enrichment analysis. **(C)** The “compound-target-pathway” network implying the mechanism of SJZD in PD treatment. The green nodes represent the compounds, the red nodes represent the targets, and the yellow nodes represent the pathways.

A total of 451 common targets between SJZD and PD were evaluated by KEGG pathway enrichment analysis (*p* < 0.05 as the significant level). Targets were highly enriched in 213 pathways (Supplementary Table S1), such as those associated with fluid shear stress and atherosclerosis, lipid and atherosclerosis, chemical carcinogenesis receptor activation, cyclic nucleotides (cAMP) signaling pathway, mitogen-activated protein kinase (MAPK) signaling pathway, AGE-RAGE signaling pathway in diabetes complications and other disease pathways. Gene counts revealed the top 20 highly enriched pathways (Figures 7A,B), suggesting that SJZD is essential for the multi-targeting and treatment of PD. To gain insight into the relationships between compounds with Degree ≥50 (Supplementary Table S2), targets, and the top 10 pathways, a “compound-target-pathway” network was created using Cytoscape 3.7.1, containing 123 nodes and 507 edges (Figure 7C).

### 3.7 Molecular docking

**FIGURE 8:**
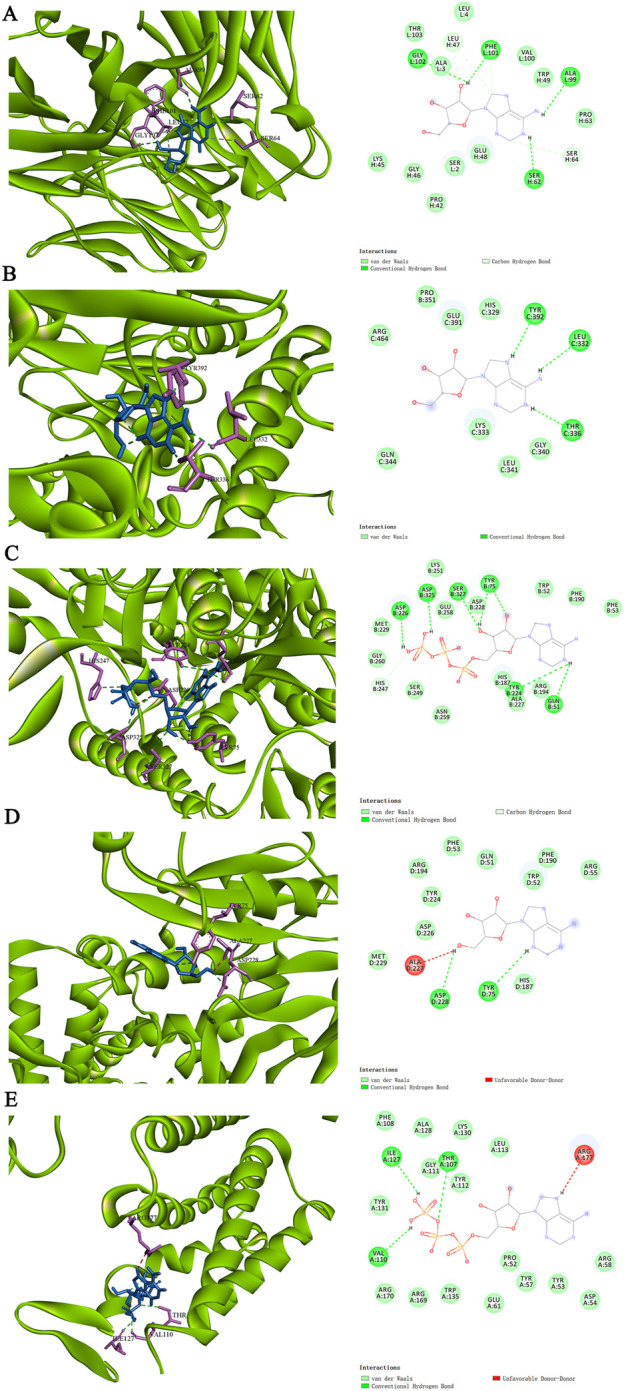
The molecular docking modes. **(A)** IL-6-Adenosine. **(B)** AKT1-Adenosine. **(C)** TNF-Adenosine Triphosphate. **(D)** TNF-Adenosine **(E)** AKT1-Adenosine Triphosphate.

Molecular docking was performed on the top five target proteins (AKT1, INS, TNF, IL-6, and TP53) and five active components (Tridecanoic Acid, Pentadecanoic Acid, Hexadecanoic Acid, Adenosine Triphosphate, and Adenosine) from the PPI and “SJZD-Active Components-Intersection Targets” network with the highest Degree values. The highest docking score (binding energy of −7.6 kcal·mol^−1^) was observed between IL-6 and Adenosine. The following highest scores were observed between AKT1 and Adenosine (−7.1 kcal·mol^−1^), TNF and Adenosine Triphosphate (−7 kcal·mol^−1^), TNF and Adenosine (−6.4 kcal·mol^−1^), and AKT1 and Adenosine Triphosphate (−6.4 kcal·mol^−1^). The results of the docking between active components and target proteins are outlined in Table 4 and illustrated in Figure 8.

**TABLE 4:** Top 10 enriched entries in GO enrichment analysis.

	Binding energy/(kcal·mol^-1^)				
Components	TP53	TNF	INS	IL6	AKT1
Tridecanoic Acid	−4	−3.7	−2.5	−2.9	−4.5
Pentadecanoic Acid	−4.5	−2.8	−2.7	−3.5	−4.4
Hexadecanoic Acid	−3.2	−2.9	−1.8	−3.3	−4.3
Adenosine Triphosphate	−5.6	−7	−4.3	−5.7	−6.4
Adenosine	−5.9	−6.7	−4.7	−7.6	−7.1

### 3.8 Molecular dynamics simulations

Molecular dynamics simulations were conducted on the top three active components with the highest absolute binding scores - compounds (IL-6-Adenosine, AKT1-Adenosine, TNF-Adenosine Triphosphate) - and the results of these simulations were used to calculate the root mean square deviation (RMSD), radius of gyration (Rog), root mean square fluctuation (RMSF), and binding free energy.

**FIGURE 9:**
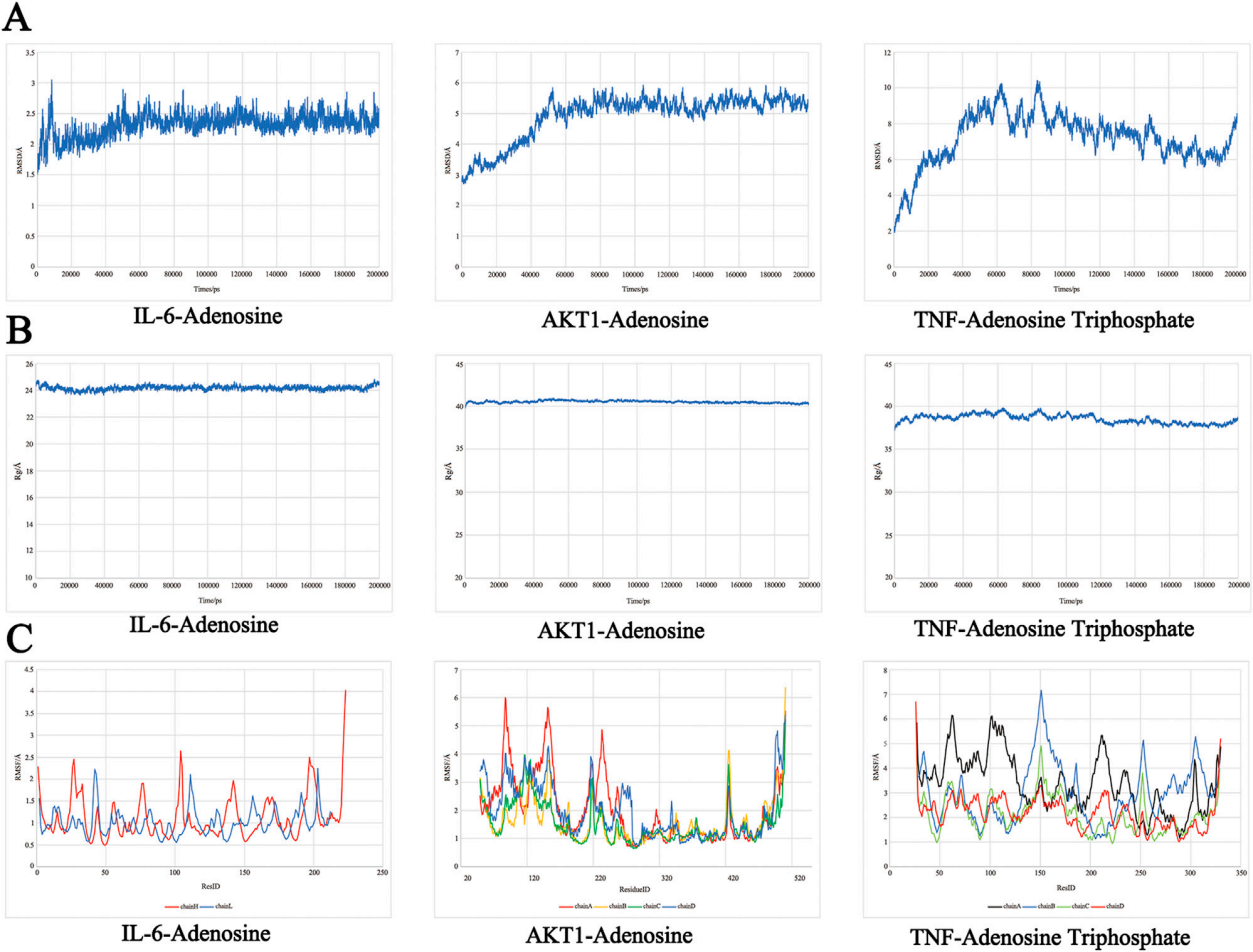
The molecular dynamics simulations. **(A)** RMSD analysis. **(B)** Rog analysis. **(C)** RMSF analysis.

The RMSD curve provides insight into the fluctuation of protein conformation (Figure 9A). The RMSD of AKT1-Adenosine protein remained relatively stable after 60 ns, stabilizing around 5Å. Similarly, the RMSD of IL6 Adenosine protein was relatively smooth, with a stable value around 2.5Å after 50 ns. These results suggest that the binding of small molecules to receptor proteins results in a stable complex system that does not cause significant changes in receptor conformation. In contrast, the RMSD of the TNF-Adenosine Triphosphate protein fluctuated significantly, demonstrating that the binding of small molecules to receptor proteins has a significant impact on protein conformation.

The Rog results (Figure 9B) demonstrated the compactness of the protein’s overall structure. The turning radii of AKT1-Adenosine and IL-6-Adenosine remained relatively stable, suggesting that the binding of small molecules to receptors did not significantly affect protein folding. On the other hand, the radius of TNF-Adenosine Triphosphate gyration exhibited some fluctuation, indicating that the binding of small molecules to receptors had a certain degree of influence on protein folding.

The RMSF curve revealed increased flexibility in certain areas of the protein core domain for AKT1-Adenosine, IL6-Adenosine, and Chain A of TNF-Adenosine Triphosphate, as demonstrated in Figure 9C. These regions were characterized by a high number of loop structures, resulting in increased flexibility compared to other sections. AKT1-Adenosine had notably higher flexibility in the 60–100 and 120–150 regions, while IL-6-Adenosine displayed increased flexibility in smaller sections of the protein core domain. Similarly, Chain A of TNF-Adenosine Triphosphate exhibited higher flexibility, particularly in the 50–130 region with a high loop content.

### 3.9 Effects of SJZD on the expression of PI3K/AKT signaling pathway proteins and apoptosis-related proteins

**FIGURE 10:**
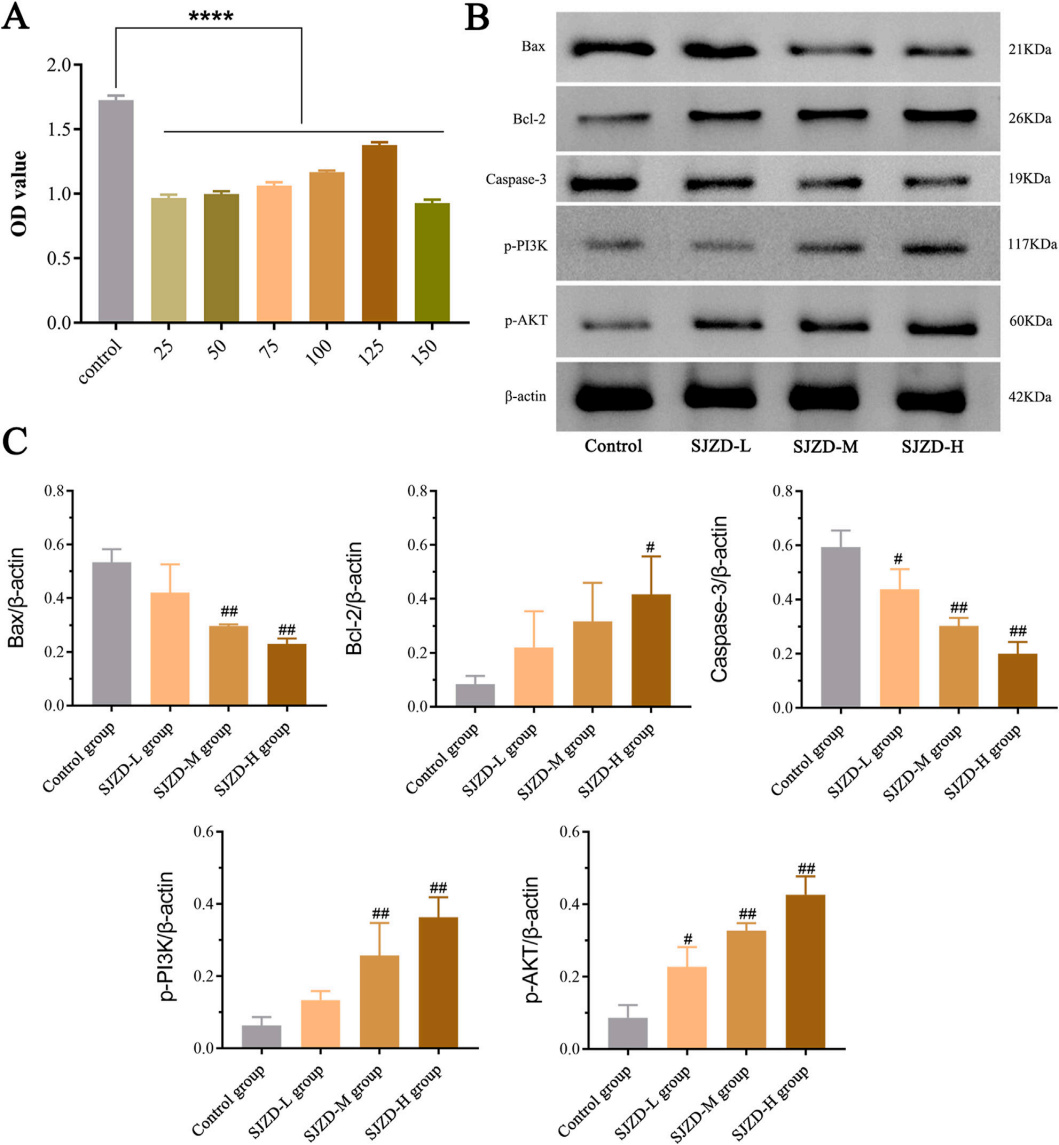
Results of cellular experiments. **(A)** The results of CCK-8 assay and the difference in absorbance value between SJZD groups. **(B)** Comparison of the expression of PI3K/AKT signaling pathway proteins and apoptosis-related proteins among all groups. **(C)** Comparison of cell protein expression among all groups. Bax/β-actin: control group vs. SJZD-M group, ^##^ *p* = 0.0012; control group vs. SJZD-H group, ^##^ *p* = 0.0006. Bal-2/β-actin: control group vs. SJZD-H group, ^#^ *p* = 0.0159. Caspase-3/β-actin: control group vs. SJZD-L group, ^#^ *p* = 0.0494; control group vs. SJZD-M group, ^##^ *p* = 0.0017; control group vs. SJZD-H group, ^##^ *p* = 0.0008. p-PI3K/β-actin: control group vs. SJZD-M group, ^##^ *p* = 0.0232; control group vs. SJZD-H group, ^##^ *p* = 0.001. p-AKT/β-actin: control group vs. SJZD-L group, ^#^ *p* = 0.0206; control group vs. SJZD-M group, ^##^ *p* = 0.0005; control group vs. SJZD-H group, ^##^ *p* = 0.0007.

CCK-8 assay demonstrated that SJZD effectively suppressed apoptosis in 6-OHDA-induced SH-SY5Y cells. Except for 150 μg/mL, SJZD groups at various concentrations (25, 50, 75, 100, and 125 μg/mL) demonstrated a notable reduction in cell apoptosis, and the rate of apoptosis was significantly lower than that of the control group (Figure 10A). WB analysis results of different SJZD concentration groups showed that the expression of p⁃PI3K and p⁃AKT in the SJZD-M group and SJZD-H group were increased significantly compared with the control group (all *p* < 0.01). However, Bax and Caspase-3 decreased significantly (all *p* < 0.01). The Bcl-2 in the SJZD-H group was increased (*p* = 0.0159), and the protein expression of cells in each group was shown in Figure 10B, and the comparison of gray value expression of cells in each group was shown in Figure 10C.

## 4 Discussion

PD is a progressive neurodegenerative disorder that predominantly affects the elderly population. This condition can substantially impact the quality of life and is linked to an increased risk of disability, as well as a potential decrease in life expectancy (Poewe et al., 2017). It has been suggested that the emergence and progression of PD are associated with various factors, such as neuroinflammation, mitochondrial dysfunction, neuronal apoptosis, and disrupted protein balance (Arena et al., 2022; Iorio et al., 2022; Li et al., 2021). Thus, suppressing the inflammatory response, oxidative stress, and apoptosis of neurons can significantly benefit PD. Recently, Chinese herbal medicine has progressed considerably in treating PD (Huang et al., 2018). Clinical studies have demonstrated that the application of SJZD and its related prescriptions is effective in treating PD (Chen et al., 2022). This study aimed to assess the target and mechanism of SJZD in treating PD via network pharmacology to evaluate its efficacy in clinical application. The findings of this research provide a significant foundation for additional investigation into the pathogenesis and treatment of PD.

### 4.1 Study on the effective components of SJZD in treating PD

This study identified 188 active components for treating PD in SJZD. The four herbs found in SJZD, namely, BaiZhu, FuLing, RenShen, and GanCao, have been proven to have therapeutic effects on PD. Ginsenosides in Ginseng, for instance, have anti-aging, antioxidant, and neuroprotective properties. It has been observed that ginsenosides can counteract the damage to the hippocampus caused by aging in mice, reduce oxidative stress levels, and downregulate the p53∼p21 signaling pathways downstream (Zhang J. J. et al., 2022). Furthermore, ginsenosides have been found to have a protective effect on dopaminergic neurons in the substantia nigra of MPTP-induced PD mice. This effect may be facilitated by γ-Aminobutyric acid neurons, which play a role in regulating the excitatory and inhibitory balance within the direct and indirect pathways associated with PD (Sharma et al., 2022; Dolgacheva et al., 2022). Research has demonstrated that Atractylodes macrocephala extract can significantly improve indicators related to oxidative stress in the brains of rats with cerebral ischemia/reperfusion. This improvement is evidenced by an increase in the activity levels of superoxide dismutase and glutathione, alongside a decrease in malondialdehyde content (Li et al., 2022). Additionally, the polysaccharides in Atractylodes macrocephala have been observed to effectively reduce apoptosis of cortical neurons in hypoxic rats. Poria cocos has also demonstrated a protective effect on the nervous system, as its extract enhances the 5-HT metabolic pathway, regulates the interaction of acetylcholine norepinephrine signal, and maintains a balanced of amino acid neurotransmitters. Additionally, it has been shown to ameliorate neurotransmitter imbalances and circadian rhythm disturbances in rats subjected to unpredictable stress (Zhang D. D. et al., 2022). Furthermore, the flavonoids, glycyrrhetinic acid, and isoliquiritigenin in licorice have been found to possess neuroprotective properties against PD caused by neurotoxins such as MPTP and rotenone (Zulfugarova et al., 2023; Moreira et al., 2023; Verma et al., 2022; Ojha et al., 2016). Moreover, studies have indicated that the flavonoid compound glycyrrhizin in licorice can promote axonal elongation of hippocampal neurons, induce the directional differentiation of neural stem cells into cholinergic neurons, and regulate ERK and AKT/GSK-3β pathways to repair nerve cell damage (Moratilla-Rivera et al., 2023; Sidiropoulou et al., 2023; Teng et al., 2014). Notably, as a TCM formula, SJZD affects multiple systems and has been documented to alleviate chronic gastritis (Wang et al., 2020) and exhibit anti-gastric cancer (Ding et al., 2022) and colorectal cancer (Shang et al., 2023).

This study also revealed that Adenosine Triphosphate, Tridecanoic Acid, Hexadecanoic Acid, Pentadecanoic Acid, and Adenosine in SJZD are the essential active components in managing PD. Adenosine Triphosphate, produced by mitochondria, has been associated with energy expenditure and calcium overload, leading to cell apoptosis, ferroptosis, and, ultimately, neuronal death in neurodegenerative diseases (Xu et al., 2022). Hexadecanoic Acid, a polyunsaturated fatty acid, has been shown to possess antioxidant and antithrombotic properties (Ganesan et al., 2022). Moreover, palmitic acid has been demonstrated to have anti-apoptotic effects by regulating the expression of B cell lymphoma 2 (Bcl-2) and activating the MAPK pathway (Kaewin et al., 2022; Wang T. et al., 2022). Adenosine is an important neuromodulator in the central nervous system involved in many brain diseases such as PD. It can influence dopaminergic signaling in the brain by interacting with its receptors to regulate cognition, pain, sleep and wakefulness, anxiety and depression, motor function, and other important physiological processes (Wang et al., 2023). Furthermore, reports show that it acts in the brain primarily through inhibitory A_1_ receptor (A_1_R) and promotive A_2A_ receptor (A_2A_R) (Fredholm et al., 2005). It has been evidenced that A_2A_R antagonists can reduce behavioral and neurochemical features of PD (Morelli et al., 2009). Therefore, the adenosine system is regarded as the primary focus of research for developing new treatments for neurological diseases.

### 4.2 Analysis of therapeutic targets of SJZD for PD

A total of 1,568 drug targets were identified for the treatment of PD in SJZD through network pharmacology analysis. Among these, five core targets were highlighted: AKT1, INS, TNF-α, IL-6, and TP53. It has been suggested that the onset of PD may be linked to the activity of AKT1, which is subject to oxidative modification that causes its dephosphorylation and subsequent neuronal degeneration in the substantia nigra of the brain (Ahmad et al., 2014). Kim et al. (2020) found that the activation of the AKT1-CREB pathway prevents neuronal cell death and motor and cognitive impairment in PD. Insulin resistance has been identified as closely related to the occurrence and development of PD, and abnormal insulin signaling pathways may lead to nervous system inflammation and mitochondrial dysfunction (Chung et al., 2022). Additionally, insulin can improve cognitive dysfunction in patients with PD and diabetes, likely due to its ability to reduce inflammation and enhance mitochondrial energy metabolism (Carvalho and Moreira, 2023). PD is characterized by the progressive loss of dopamine (DA) neurons in the substantia nigra pars compacta (SNpc) (Balestrino and Schapira, 2020), and evidence suggests that neuroinflammation plays an important role in the pathological features and symptoms of PD (Zhang et al., 2023). It has been noted that the serum of PD patients contains elevated levels of TNF-α and IL-6 compared to that of healthy individuals (Shamsdin et al., 2022; Li et al., 2023a; Moradi et al., 2023; Qu et al., 2023). TNF-α has been shown to significantly regulate modifications of the nigrostriatal pathway and to facilitate the apoptosis of dopaminergic neurons, particularly in chronic inflammation (Amin et al., 2022). Furthermore, TNF-α can activate Caspase-mediated cell apoptosis, thus exacerbating the progression of the disease (Coles et al., 2018). Therefore, targeting TNF-α allow for the optimization of its dual neurotoxic and neuroprotective effects in PD. Notably, a recent study (Green et al., 2019) found that IL-6 was positively associated with motor scores in male patients, while higher levels of IL-6 were associated with poorer cognitive performance in female patients. Another study (Fu et al., 2023) also reported a correlation between IL-6 levels and non-motor symptoms and cognitive dysfunction in PD patients, suggesting that IL-6 may be involved in the pathophysiological processes of non-motor symptoms in PD patients. Moreover, TP53/P53 is a transcription factor that regulates cell growth, proliferation, and repair. In response to stress, TP53 may act on signaling pathways such as the Bcl-2 family protein phase or MAPK, thereby playing a role in modulating cell apoptosis and inflammatory response (Dai et al., 2016; Su et al., 2022).

### 4.3 Analysis of PD signaling pathway treated by SJZD

KEGG pathway enrichment analysis revealed that SJZD treatment of PD primarily targets the Dopaminergic synapse, Lipid and atherosclerosis, cAMP signaling pathway, glycosylated protein products (AGEs) and their ligand (RAGE) pathway in diabetic complications, MAPK signaling pathway, and other signaling pathways. Research has suggested that the lack of dopaminergic synapses in the ventral tegmental area of PD patients may lead to depressive symptoms, while synaptic plasticity damage in the striatum may reduce dopamine levels and trigger the onset of the disease (Cantello et al., 1989). Furthermore, inflammation of peripheral organs has been linked to the exacerbation of the progression of PD (Zhang et al., 2023). cAMP is of great importance in the central nervous system cells, playing a role in neuron growth, development, cognition, and the regulation of dendritic and axonal growth (Zhou et al., 2019). Wu et al. (2018) found that by inhibiting miRNA-200a, the expression of dopamine receptor D2 in the striatum is increased, and apoptosis in the striatum of PD rats is suppressed via the cAMP/PKA pathway, thus improving the symptoms of the disease. Another study has also demonstrated that AGEs-RAGE pathways can contribute to the development of PD, with their mechanism of action associated with inflammatory response (Naz et al., 2023). MAPK signaling pathways are also implicated in the initiation and progression of cell apoptosis. ERK1/2, the most studied member of the MAPK family, is thought to induce cell apoptosis by causing the release of cytochrome C, decreasing the anti-apoptotic protein Bcl-2, increasing the pro-apoptotic protein Bax, and activating the apoptotic gene Caspase-9, leading to a cascade reaction and ultimately activating Caspase-3 (Rivo et al., 2007).

### 4.4 Analysis of molecular docking and molecular dynamics simulation results

The molecular docking results of this study suggest that IL-6 and Adenosine have the strongest binding affinity, with a binding energy of −7.6 kcal·mol^−1^. This is followed by AKT1 and Adenosine (−7.1 kcal·mol^−1^), TNF-Adenosine Triphosphate (−7 kcal·mol^−1^), TNF-Adenosine (−6.4 kcal·mol^−1^), and AKT1-Adenosine Triphosphate (−6.4 kcal·mol^−1^). These findings indicate that the core active components can form a stable ligand-receptor complex system with the target proteins, thus playing a significant role in treating PD. Furthermore, the molecular dynamics simulation results show that the RMSD and Rog of AKT1-Adenosine and IL-6-Adenosine are relatively gentle, implying that the formation of the complex will not cause significant changes in receptor conformation and protein folding. On the other hand, the RMSD and Rog fluctuations of TNF-Adenosine Triphosphate are substantial, indicating that the formation of the complex has an impact on protein conformation and protein folding. In addition, it is worth mentioning that a recent study (Li et al., 2024) found that elevated levels of the pro-inflammatory factor IL-6 can activate the PI3K-AKT pathway.

Studies have shown that the PI3K/AKT pathway is mainly involved in regulating various biological functions such as cell proliferation, cell differentiation, and apoptosis (Thorpe et al., 2015). Of consequence, apoptosis and oxidative stress have been reported to play an important role in the pathogenesis of PD, and inhibiting apoptosis can effectively alleviate PD symptoms (Trist et al., 2019). Recently, research has revealed the significance of activating the PI3K/AKT pathway and its neuroprotective value in PD (Yao et al., 2022; Li et al., 2023b). The PI3K/AKT cascade, which acts on a large number of proteins, is considered a key player in PD models, as research indicates its ability to significantly improve neuronal survival, promote neurogenesis, and inhibit neurotoxin-induced apoptosis (Zheng M. et al., 2021; Khezri and Ghasemnejad-Berenji, 2022; Zheng X. et al., 2021; Wang Q. et al., 2022). Therefore, apoptosis, the most widely explored mechanism of PD neuron loss, was selected for study in this experiment. The results showed that after SJZD intervention, the expressions of PI3K/AKT signaling pathway proteins (p-PI3K, p-AKT) and anti-apoptotic protein (Bcl-2) both increased. Furthermore, it was observed that the expression of these proteins increased in accordance with the concentration of SJZD administered. The higher the expression of the related protein, the stronger the inhibitory impact of SJZD on apoptosis.

This study employed a combination of network pharmacology, molecular docking, and molecular dynamics simulation techniques to construct an “SJZD-Active Components-Intersection Targets” network and PPI network to identify the core active components and action targets of SJZD and to investigate its potential mechanism of action in treating PD. It should be noted that the study has some limitations. First, bioactive compounds and target data were obtained through literature and databases to facilitate predictions, with the reliability and accuracy of these predictions being contingent upon the quality of the data. Future studies can address this issue through liquid chromatography/mass spectrometry (LC/MS) technique, both for the analysis of the active compounds of SJZD, and for the study of metabolomics and pharmacokinetic. Secondly, due to the limitations of network pharmacology, the mechanism of SJZD treatment of PD has only been studied through data mining, and there is a lack of PD animal models and *in vivo* analyses. Thus, clinical trials and animal experiments are still needed to verify the efficacy and mechanism of SJZD in the treatment of PD. Moreover, the binding sites of SJZD with key targets will become the focus of our subsequent studies.

## 5 Conclusion

SJZD alleviates apoptosis by activating in the PI3K/AKT signaling pathway, which is consistent with the predictions of network pharmacology, molecular docking, and molecular dynamics simulations. This study provides valuable information on the active elements, targets, and action mechanisms of SJZD. This study further established that AKT1, IL-6, and the P13K/AKT pathways are the key targets of SJZD in the treatment of PD. It also identified the optimal dosage of SJZD, which can be utilized to investigate its potential applications in PD and the effectiveness of spleen-invigorating treatments for PD, thereby offering valuable insights for clinical accuracy.

## Data Availability

The datasets presented in this study can be found in online repositories. The names of the repository/repositories and accession number(s) can be found in the article.
