# Supplementary figures and images for: Molecular targets and mechanisms of Sijunzi decoction in the treatment of Parkinson’s disease: evidence from network pharmacology, molecular docking, molecular dynamics simulation, and experimental validation
Source: Front Pharmacol. 2024 Nov 26;15:1487474. doi: 10.3389/fphar.2024.1487474 (PMC11629541; doi:10.3389/fphar.2024.1487474)

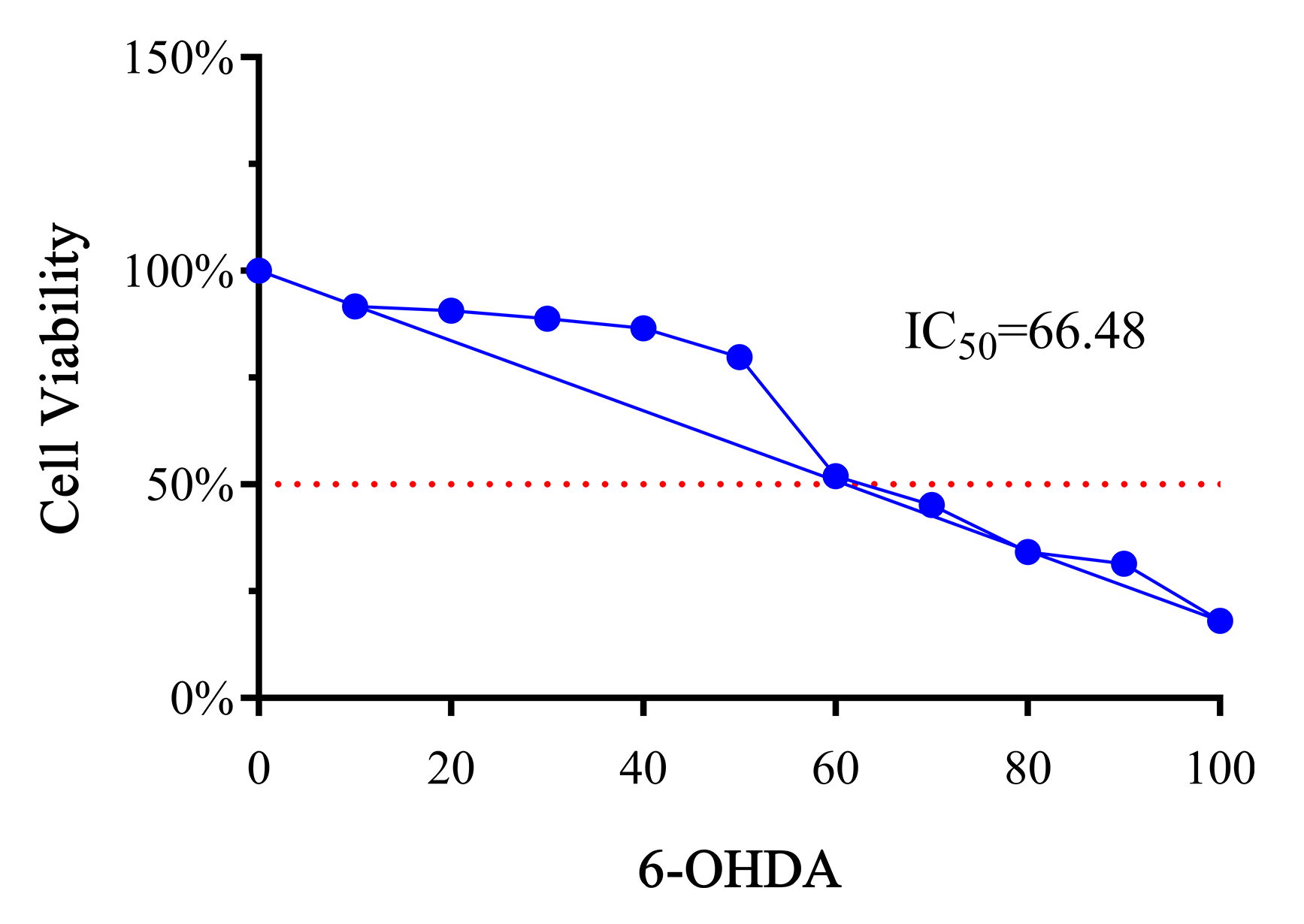

Supplement: Supplementary file 2 [file Image1.TIF]
